# Hypoxia-preconditioned mesenchymal stem cells prevent renal fibrosis and inflammation in ischemia-reperfusion rats

**DOI:** 10.1186/s13287-020-01642-6

**Published:** 2020-03-20

**Authors:** Naoki Ishiuchi, Ayumu Nakashima, Shigehiro Doi, Ken Yoshida, Satoshi Maeda, Ryo Kanai, Yumi Yamada, Takeshi Ike, Toshiki Doi, Yukio Kato, Takao Masaki

**Affiliations:** 1 Department of Nephrology, Hiroshima University Hospital, 1-2-3 Kasumi, Minami-ku, Hiroshima, 734-8551 Japan; 2 Department of Stem Cell Biology and Medicine, Graduate School of Biomedical & Health Sciences, Hiroshima University, 1-2-3 Kasumi, Minami-ku, Hiroshima, 734-8553 Japan; 3 TWOCELLS Company, Limited, 16-35 Hijiyama-honmachi, Minami-ku, Hiroshima, 732-0816 Japan

**Keywords:** Mesenchymal stem cells, Hypoxia, Vascular endothelial growth factor, Hepatocyte growth factor, Renal fibrosis

## Abstract

**Background:**

Mesenchymal stem cells (MSCs) have been reported to promote the regeneration of injured tissue via their paracrine abilities, which are enhanced by hypoxic preconditioning. In this study, we examined the therapeutic efficacy of hypoxia-preconditioned MSCs on renal fibrosis and inflammation in rats with ischemia-reperfusion injury (IRI).

**Methods:**

MSCs derived from rats and humans were incubated in 1% O_2_ conditions (1%O_2_ MSCs) for 24 h. After IRI, 1%O_2_ MSCs or MSCs cultured under normoxic conditions (21%O_2_ MSCs) were injected through the abdominal aorta. At 7 or 21 days post-injection, the rats were sacrificed and their kidneys were analyzed. In in vitro experiments, we examined whether 1%O_2_ MSCs enhanced the ability to produce anti-fibrotic humoral factors using transforming growth factor (TGF)-β1-stimulated HK-2 cells incubated with conditioned medium from MSCs.

**Results:**

Administration of rat 1%O_2_ MSCs (1%O_2_ rMSCs) attenuated renal fibrosis and inflammation more significantly than rat 21%O_2_ MSCs. Notably, human 1%O_2_ MSCs (1%O_2_ hMSCs) also attenuated renal fibrosis to the same extent as 1%O_2_ rMSCs. Flow cytometry showed that 1%O_2_ hMSCs did not change human leukocyte antigen expression. Further in vitro experiments revealed that conditioned medium from 1%O_2_ MSCs further suppressed TGF-β1-induced fibrotic changes in HK-2 cells compared with 21%O_2_ MSCs. Hypoxic preconditioning enhanced vascular endothelial growth factor (VEGF) and hepatocyte growth factor (HGF) secretion. Interestingly, VEGF knockdown in 1%O_2_ MSCs attenuated HGF secretion and the inhibition of TGF-β1-induced fibrotic changes in HK-2 cells. In addition, VEGF knockdown in 1%O_2_ hMSCs reduced the anti-fibrotic effect in IRI rats.

**Conclusions:**

Our results indicate that hypoxia-preconditioned MSCs are useful as an allogeneic transplantation cell therapy to prevent renal fibrosis and inflammation.

## Background

The prevalence of chronic kidney disease (CKD) is estimated to be 10–15% worldwide [1, 2]. Therefore, it is currently recognized as a world health concern with evidence that CKD patients have an increased risk of not only cardiovascular diseases, but also all-cause mortality [3, 4]. In clinical settings, CKD occurs in the elderly and patients with chronic diseases that cause renal damage, such as chronic glomerular nephritis, hypertension, and diabetes mellitus [5–7]. On the other hand, acute kidney injury (AKI) is defined as an abrupt decrease in renal function, and most patients should recover to their baseline level after AKI. However, recent studies have revealed that a significant number of AKI patients eventually develop CKD [8–10], so called “AKI to CKD transition.” Thus, AKI has attracted attention as a novel risk factor of CKD.

Pathologically, interstitial fibrosis and inflammation are common features in CKD patients regardless of primary disease [11, 12]. Although ischemia-reperfusion injury (IRI) is a well-established rodent model of AKI, progression of renal fibrosis and inflammation are also observed in the kidney after IRI [13]. According to previous studies, several pathological mechanisms, such as hypoxia, microvascular rarefaction, inflammation, transforming growth factor (TGF)-β1 production, and epithelial-mesenchymal transition (EMT), reportedly participate in AKI to CKD transition [14–16]. However, there are currently no effective therapies to prevent AKI to CKD progression.

Some studies have reported that the administration of mesenchymal stem cells (MSCs) exerts beneficial effects against various diseases such as heart disease [17], stroke [18], and autoimmune disease [19]. MSCs were previously considered to promote the regeneration of injured tissue by their capacity to differentiate into multiple tissue types [20, 21]. However, rather than differentiating, paracrine activities are likely implicated in the therapeutic effects of MSCs [22, 23]. Notably, recent studies have reported that hypoxia-preconditioned MSCs intensify their paracrine abilities [24, 25]. These findings led us to the hypothesis that MSCs cultured under 1% O_2_ conditions (1%O_2_ MSCs) suppress IRI-induced renal fibrosis and inflammation more strongly than MSCs cultured under normoxic conditions (21%O_2_ MSCs).

In this study, using rat- and human-derived bone marrow MSCs, we show that 1%O_2_ MSCs ameliorate renal fibrosis and inflammation in vivo and in vitro. We also show that vascular endothelial growth factor (VEGF), hepatocyte growth factor (HGF), and prostaglandin E2 (PGE2) increase in medium of 1%O_2_ MSCs compared with that of 21%O_2_ MSCs. In addition, we show that inhibition of VEGF suppresses HGF secretion and the anti-fibrotic effect of 1%O_2_ MSCs. These findings suggest that the administration of hypoxia-preconditioned MSCs may be a candidate therapy to prevent renal fibrosis and inflammation after AKI.

## Methods

### Animals

Male Sprague-Dawley (SD) rats (6 and 8 weeks old) were purchased from Charles River Laboratories Japan (Yokohama, Japan). The rats at 6 weeks of age were used to collect bone marrow, and rats at 8 weeks were used to induce IRI. Male CAG-enhanced green fluorescent protein (EGFP)-transgenic SD rats (6 weeks old) were purchased from Japan SLC (Shizuoka, Japan) to collect bone marrow. All experimental procedures were approved by the Institutional Animal Care and Use Committee of Hiroshima University (Hiroshima, Japan) (permit number, A16-83) and conducted in accordance with the *Guide for the Care and Use of Laboratory Animals*, *8th ed*, 2010 (National Institutes of Health, Bethesda, MD, USA).

### Preparation of MSCs

According to previously described methods [26], bone marrow was collected from SD and CAG-EGFP-transgenic SD rats and cultured in standard culture medium consisting of DMEM (Sigma-Aldrich, St. Louis, MO, USA) with 10% FBS (Sigma-Aldrich). Cells were passaged four times and used as rat MSCs for transplantation. Human MSCs from bone marrow were purchased from Riken BRC (Ibaraki, Japan). These cells were also cultured in DMEM containing 10% FBS.

### Hypoxic preconditioning

To perform hypoxic preconditioning, MSCs were cultured in DMEM with 10% FBS. At 80% confluence, fresh complete medium was added and hypoxic preconditioning was performed with a Modular Incubator Chamber (MIC 101) (Billups-Rothenberg, San Diego, CA, USA). The cells were incubated in hypoxic conditions (1% O_2_) for 24 h.

### Experimental animal model

Renal IRI was performed by transiently clamping the unilateral renal artery. Rats were anesthetized by an intraperitoneal injection of three types of mixed anesthetic agents: medetomidine, midazolam, and butorphanol. After a laparotomy was performed, the left kidney was exposed. Then, the renal pedicle was clamped by atraumatic vascular clamps for 1 h, followed by reperfusion on a heating blanket. After reperfusion, MSCs (5 × 10^5^ cells/rat) in 0.2 ml PBS were injected through the abdominal aorta clamped above and below the left renal artery bifurcation. At 7 or 21 days post-injection, the rats were sacrificed and their kidneys were collected.

### Western blot analysis

Sample collection and western blotting were performed according to previously described methods [26]. Mouse monoclonal anti-α-SMA antibody (Sigma-Aldrich), mouse monoclonal anti-TGF-β1 antibody (Santa Cruz Biotechnology, Santa Cruz, CA, USA), mouse monoclonal anti-GAPDH antibody (Sigma-Aldrich), rabbit monoclonal anti-p-Smad2 antibody (Cell Signaling Technology, Danvers, MA, USA), mouse monoclonal anti-Smad2 antibody (Cell Signaling Technology), and mouse monoclonal anti-α-Tubulin antibody (Sigma-Aldrich) were used as primary antibodies. Horseradish peroxidase-conjugated goat anti-rabbit immunoglobulin G (Dako, Glostrup, Denmark) or goat anti-mouse immunoglobulin G (Dako) was used as secondary antibodies. SuperSignal West Dura or the Pico system (Thermo Fisher Scientific, Rockford, IL, USA) were used to detect signals. The intensity of each band was measured by ImageJ software (version 1.47v; National Institutes of Health) and normalized to the level of either GAPDH or α-tubulin.

### Immunohistochemistry analysis

Immunohistochemical staining was performed according to previously described methods [26]. The following primary antibodies were used: mouse monoclonal anti-α-SMA antibody (Sigma-Aldrich), rabbit polyclonal anti-collagen type I antibody (Abcam, Cambridge, UK), rabbit polyclonal anti-collagen type III antibody (Abcam), rabbit polyclonal anti-CD3 antibody (Dako), rabbit polyclonal anti-CD68 antibody (Abcam), rabbit monoclonal anti-CD163 antibody (Abcam), and mouse monoclonal anti-EGFP antibody (Takara Bio, Shiga, Japan). CD3-, CD68-, and CD163-positive cells and positive areas for α-SMA and collagen type I and III staining were assessed using ImageJ software by examination of five randomly selected fields (× 100) of the cortex.

### Immunohistochemistry analysis (double immunostaining)

Sections of formalin-fixed, paraffin-embedded tissues (4 μm thick) were de-paraffinized, subjected to heat-mediated antigen retrieval in EDTA buffer (pH 9.0) at 98 °C for 40 min, and then blocked in 2.5% normal horse serum (ImmPRESS Horse Anti-Rabbit IgG Polymer kit; Vector Laboratories, Riverside, CA, USA) at room temperature for 20 min. They were incubated with anti-CD163 antibody (Abcam) overnight at 4 °C, followed by incubation with the appropriate secondary antibody (ImmPRESS Horse Anti-Rabbit IgG Polymer kit; Vector Laboratories) at room temperature for 30 min, and then incubated with 3,3′-diaminobenzidine (Sigma-Aldrich) at room temperature for 5 min. After that, they were heated again in EDTA buffer in the same way. They were then blocked, followed by incubation with anti-CD68 antibody (Abcam) and the secondary antibody in the same way, and next incubated with working solution prepared with Vector SG Peroxidase (HRP) Substrate Kit (Vector Laboratories) at room temperature for 5 min.

### Histological analysis

Sections of formalin-fixed, paraffin-embedded tissues (2 μm thick) were stained with hematoxylin and eosin (HE), Masson trichrome, and Sirius red to assess histological injury and fibrosis. The areas of interstitial fibrosis were assessed using Lumina Vision (Mitani, Osaka, Japan) by examining five randomly selected fields (× 100) of the cortex.

### MSC labeling

Human MSCs were labeled with CellTracker CM-DiI (Thermo Fisher Scientific) before injection, following the manufacturer’s protocols.

### Flow cytometric analysis

Flow cytometric analysis was performed according to previously described methods [26]. The following antibodies were used: anti-human CD29 IgG antibody (BioLegend, San Diego, CA, USA), anti-human CD44 IgG antibody (BioLegend), anti-human CD73 IgG antibody (BioLegend), anti-human CD90 IgG antibody (BioLegend), anti-human CD11b IgG antibody (BioLegend), anti-human CD34 IgG antibody (BioLegend), anti-human CD45 IgG antibody (BioLegend), anti-human HLA-A,B,C IgG antibody (BioLegend), and anti-human HLA-DR IgG antibody (BioLegend). The stained MSCs were analyzed using a BD FACSVerse (Becton, Dickinson and Company, Franklin Lakes, NJ, USA). Data were assessed by FlowJo software (FlowJo, LLC; Ashland, OR, USA).

### Preparation of conditioned medium

To generate conditioned medium from human 1%O_2_ MSCs (1%O_2_ hMSCs-CM) and human 21%O_2_ MSCs (21%O_2_ hMSCs-CM), human MSCs (3 × 10^5^ cells/dish) were seeded into 10-cm dishes and grown to 80% confluence. The culture medium was then substituted with DMEM containing 0.1% FBS, and the cells were cultured for 24 or 48 h under hypoxic (1% O_2_) or normoxic (21% O_2_) conditions. Then, each medium was collected.

### Cell culture and treatments

HK-2 cells were obtained from the American Type Culture Collection (Manassas, VA, USA). The cells were cultured as described previously [26]. After the starvation of HK-2 cells with conditioned medium from human MSCs or DMEM containing 0.1% FBS for 24 h, 10 ng/ml recombinant human TGF-β1 (R&D Systems, Minneapolis, MN, USA) was added to the cells directly. After 30 min (to investigate protein levels of p-Smad 2) or 48 h (to investigate protein levels of α-SMA), HK-2 cells were collected and subjected to in vitro study.

### Counting MSCs

Human MSCs (5 × 10^4^ cells/well) were seeded into six-well plates and grown to 60–80% confluence. The culture medium was then substituted with fresh DMEM containing 0.1% or 10% FBS, and the cells were cultured under hypoxic (1% O_2_) or normoxic (21% O_2_) conditions. The number of these cells was counted after 24 or 48 h.

### Quantitative real-time reverse-transcription PCR

RNA extraction and real-time reverse-transcription PCR were performed in accordance with previously described methods [26]. Specific oligonucleotide primers and probes for VEGF (assay ID Hs00900055_m1), HGF (assay ID Hs00300159_m1), and 18S rRNA (endogenous control) were obtained as TaqMan Gene Expression Assays (Applied Biosystems, Foster City, CA, USA). The mRNA levels were standardized by the level of 18S rRNA.

### ELISAs

ELISA analyses of VEGF (R&D Systems), HGF (R&D Systems), and PGE2 (Enzo Life Science, Farmingdale, NY, USA) were performed following the manufacturers’ protocols. Concentrations were normalized to the total protein content.

### Transfection of VEGF siRNA

Human MSCs were transfected with 20 nM siRNA targeting VEGF (sc-461; Applied Biosystems) or negative control siRNA (4390843; Applied Biosystems) using Lipofectamine 2000 Transfection Reagent (Thermo Fisher Scientific). After 24 h, the transfected cells were washed, and fresh complete medium was added. At 80% confluence, hypoxic preconditioning was performed. Then, the cells were collected and injected into the abdominal aorta. On the other hand, to generate conditioned medium from human 1%O_2_ MSCs transfected with VEGF siRNA or negative control siRNA, at 80% confluence, the transfected cells were cultured in DMEM containing 0.1% FBS for 48 h under hypoxic (1% O_2_) conditions. Then, each medium was collected.

### Statistical analysis

Results are expressed as means ± standard deviations (S.D.). For multiple group comparisons, one-way ANOVA followed by Bonferroni’s post hoc test was applied. Comparisons between two groups were analyzed by the Student’s *t* test. *P* < 0.05 was considered as statistically significant.

## Results

### Hypoxia-preconditioned rat MSCs attenuate IRI-induced renal fibrosis in rats

**Fig. 1:**
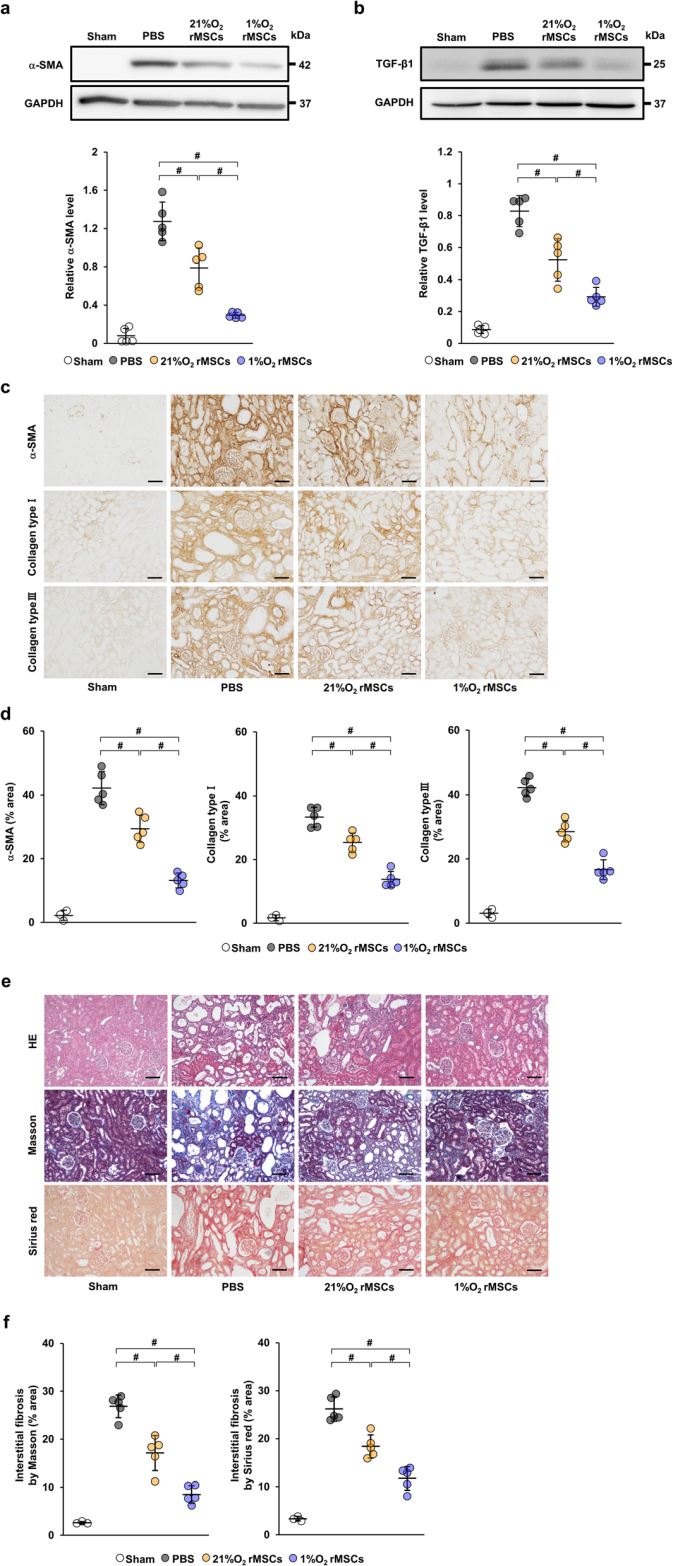
1%O_2_ rMSCs suppress renal fibrosis more significantly than 21%O_2_ rMSCs in IRI rats. **a**, **b** Western blot analysis of α-SMA and TGF-β1 in the kidney cortex of IRI rats at day 21 post-IRI. Graphs show densitometric analyses of α-SMA and TGF-β1 expression levels normalized to the GAPDH expression level. **c** Representative immunohistochemical staining of α-SMA and collagen type I and III in kidney sections at day 21 post-IRI (scale bar = 100 μm). **d** Quantification of α-SMA- and collagen type I- and III-positive areas as percentages of the total area. **e** Representative images of HE, Masson trichrome, and Sirius red staining in kidney sections at day 21 post-IRI (scale bar = 100 μm). **f** Quantification of interstitial fibrosis area as percentages of the total area. Data are means ± S.D. ^#^*P* < 0.01 (one-way ANOVA followed by Bonferroni’s post hoc test)

To investigate the effect of hypoxia-preconditioned rat MSCs on renal fibrosis, we first examined the expression of α-SMA and TGF-β1 in the IRI model that had been injected with PBS, 5 × 10^5^ rat MSCs cultured under normoxic conditions (21%O_2_ rMSCs), or under 1% O_2_ conditions (1%O_2_ rMSCs) at 21 days post-IRI. As shown in Fig. 1a and b, protein levels of α-SMA and TGF-β1 were remarkably increased in IRI rats injected with PBS (PBS group). Such expression was suppressed in IRI rats injected with 21%O_2_ rMSCs (21%O_2_ rMSC group), and further suppression was observed in those injected with 1%O_2_ rMSCs (1%O_2_ rMSC group). Similarly, immunostaining revealed that the α-SMA-positive area was reduced in the 1%O_2_ rMSC group compared with the 21%O_2_ rMSC group (Fig. 1c, d). Furthermore, immunostaining revealed that collagen type I- and III-positive areas were more significantly suppressed in the 1%O_2_ rMSC group than in the 21%O_2_ rMSC group (Fig. 1c, d). Moreover, we performed HE staining at 7 and 21 days post-IRI. HE staining showed tubular dilatation, tubular cast formation, and diffused infiltration of inflammatory cells at 7 days post-IRI. These tubulointerstitial injuries were suppressed by the administration of 21%O_2_ rMSCs or 1%O_2_ rMSCs, with no significant difference between them (Additional file 1). Further progression of tubulointerstitial injuries and tubular atrophy were found at 21 days post-IRI. The progression of tubulointerstitial injuries and tubular atrophy were suppressed by the administration of 21%O_2_ rMSCs, and further suppression was observed in the 1%O_2_ rMSC group (Fig. 1e). Masson trichrome staining and Sirius red staining showed only slight interstitial fibrosis at 7 days post-IRI (Additional file 1), so we considered that the rat model at 7 days post-IRI was not suitable for evaluating the anti-fibrotic effect of MSCs. At 21 days post-IRI, further progression of interstitial fibrosis was observed in the PBS group and was attenuated by MSC treatment, particularly in the 1%O_2_ rMSC group (Fig. 1e, f).

### Hypoxia-preconditioned rat MSCs suppress the infiltration of inflammatory cells in IRI rats

**Fig. 2:**
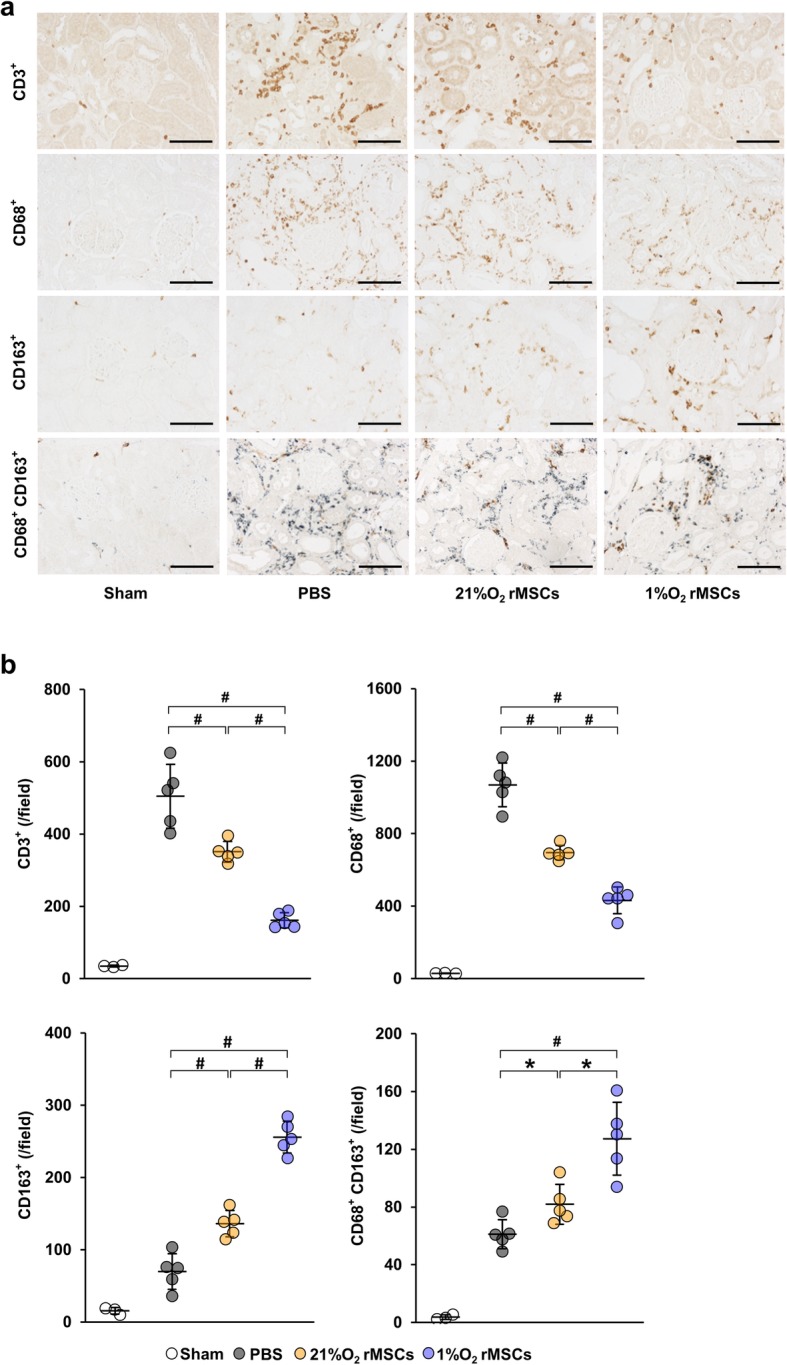
1%O_2_ rMSCs attenuate renal inflammation more strongly than 21%O_2_ rMSCs in IRI rats. **a** Representative images of immunostaining of CD3, CD68, and CD163 and double immunostaining of CD163 (brown) and CD68 (blue) in the kidney sections at day 7 post-IRI (scale bar = 100 μm). **b** Quantification of CD3-, CD68-, and CD163-positive cells and double-positive (CD68 and CD163) cells. Data are means ± S.D. ^#^*P* < 0.01, *P < 0.05 (one-way ANOVA followed by Bonferroni’s post hoc test)

To assess the anti-inflammatory effect of hypoxia-preconditioned rat MSCs, we examined the expression of CD3 (T cell marker), CD68 (M1 and M2 macrophage marker), and CD163 (M2 macrophage marker) at 7 days post-IRI. Immunohistochemical staining revealed that the accumulation of CD3- and CD68-positive cells was increased in the PBS group (Fig. 2a, b). The infiltration of these cells was suppressed in the 21%O_2_ rMSC group, whereas it was suppressed more significantly in the 1%O_2_ rMSC group (Fig. 2a, b). In contrast, the expression of CD163, an immunosuppressive macrophage marker, was increased in the 21%O_2_ rMSC group, and a further increase was observed in the 1%O_2_ rMSC group (Fig. 2a, b). In addition, we performed double immunostaining for CD68 and CD163 to identify M2 macrophages. Positive cells for both CD68 and CD163 were increased in the 21%O_2_ rMSC group, and a further increase was observed in the 1%O_2_ rMSC group (Fig. 2a, b).

### Hypoxia-preconditioned human MSCs also attenuate renal fibrosis in IRI rats

**Fig. 3:**
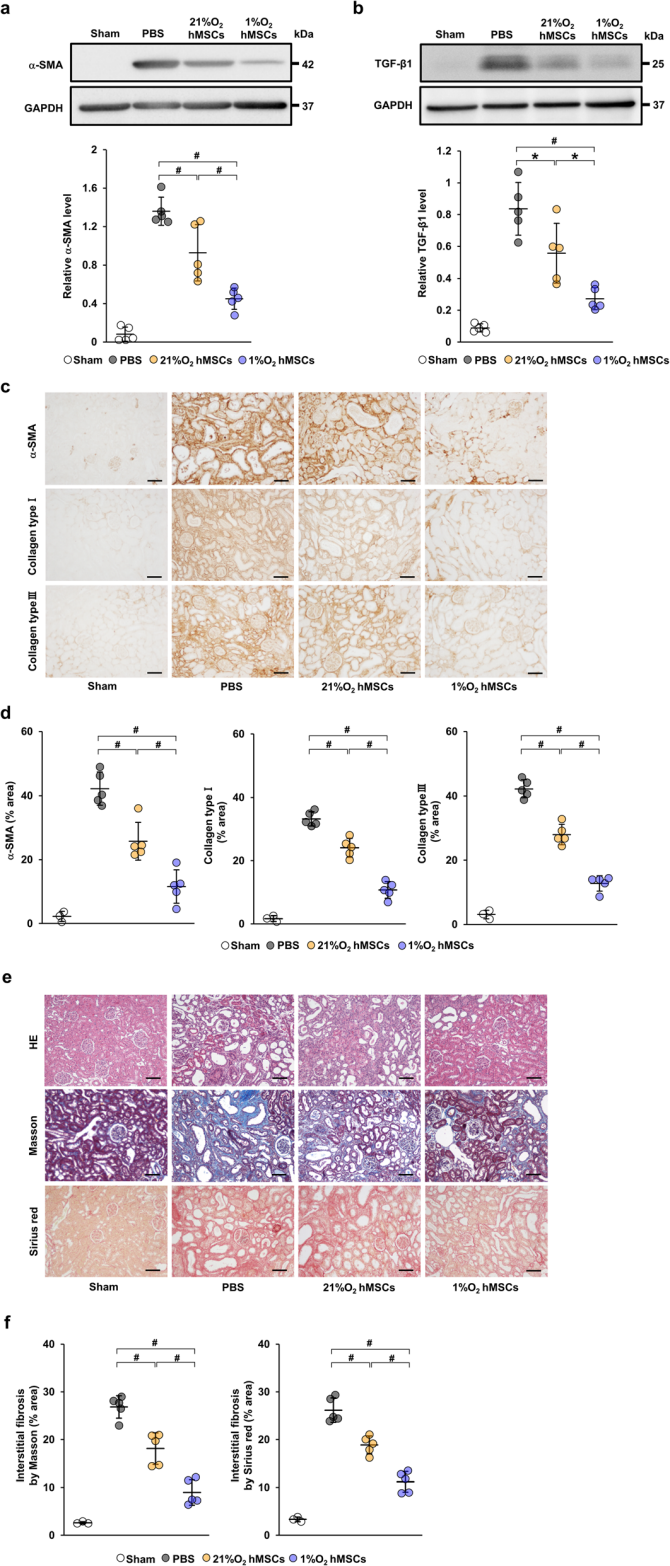
1%O_2_ hMSCs suppress renal fibrosis more significantly than 21%O_2_ hMSCs in IRI rats. **a**, **b** Western blot analysis of α-SMA and TGF-β1 in the kidney cortex of IRI rats at day 21 post-IRI. Graph shows densitometric analysis of α-SMA and TGF-β1 expression levels normalized to the GAPDH expression level. **c** Representative immunohistochemical staining of α-SMA and collagen type I and III in kidney sections at day 21 post-IRI (scale bar = 100 μm). **d** Quantification of α-SMA- and collagen type I- and III-positive areas as percentages of the total area. **e** Representative images of HE, Masson trichrome, and Sirius red staining in kidney sections at day 21 post-IRI (scale bar = 100 μm). **f** Quantification of interstitial fibrosis area as percentages of the total area. Data are means ± S.D. ^#^*P* < 0.01, *P < 0.05 (one-way ANOVA followed by Bonferroni’s post hoc test)

To investigate whether hypoxia-preconditioned human MSCs also exerted therapeutic effects on renal fibrosis, we next injected PBS, 5 × 10^5^ human MSCs cultured under normoxic conditions (21%O_2_ hMSCs), or under 1% O_2_ conditions (1%O_2_ hMSCs) into the IRI model (21%O_2_ hMSC and 1%O_2_ hMSC groups, respectively). Although the upregulation of α-SMA and TGF-β1 in the PBS group was suppressed in the 21%O_2_ hMSC group, their expression in the 1%O_2_ hMSC group was reduced more significantly than in the 21%O_2_ hMSC group at 21 days post-IRI (Fig. 3a, b). Similarly, immunostaining revealed that the α-SMA-positive area was reduced in the 1%O_2_ hMSC group compared with the 21%O_2_ hMSC group (Fig. 3c, d). Furthermore, immunostaining revealed that collagen type I- and III-positive areas were more significantly suppressed in the 1%O_2_ hMSC group than in the 21%O_2_ hMSC group (Fig. 3c, d). Moreover, HE staining showed that tubulointerstitial injuries were more significantly suppressed in the 1%O_2_ hMSC group than in the 21%O_2_ hMSC group (Fig. 3e). Masson trichrome staining and Sirius red staining revealed that the area of interstitial fibrosis was reduced by MSC treatment, particularly in the 1%O_2_ hMSC group (Fig. 3e, f).

### Hypoxic preconditioning does not change the immunophenotype of MSCs

It has been reported that the preconditioning of MSCs with hypoxia enhanced their migration capacity [25, 27]. Therefore, we studied whether hypoxic preconditioning enhances the engraftment capacity of MSCs using MSCs from male CAG-EGFP-transgenic SD rats. EGFP-positive cells were assessed as the average of ten randomly selected fields (× 100) for each rat at 21 days post-IRI (1%O_2_ rMSC group and 21%O_2_ rMSC group, *n* = 5). Immunohistochemical staining for EGFP revealed that the number of EGFP-positive cells was 10 ± 1 cells/10 fields in the 1%O_2_ rMSC group and 10 ± 1 cells/10 fields (× 100) in the 21%O_2_ rMSC group (Additional file 2a). These results suggest that hypoxic preconditioning does not increase the engraftment capacity of MSCs. In contrast, we found that anti-fibrotic ability was almost equal in 1%O_2_ rMSCs and 1%O_2_ hMSCs. Therefore, we investigated their engraftment using rMSCs from EGFP-transgenic rats and DiI-labeled hMSCs. EGFP-expressing rMSCs and DiI-labeled hMSCs were both observed in the kidney at day 21 post-IRI (Additional file 2a, b). Next, to assess whether hypoxic preconditioning affected their immunophenotype, we analyzed expression levels of cell surface markers using flow cytometry. Although human leukocyte antigen (HLA) expression plays an important role in the allogeneic immune response, the expression level of HLA-A,B,C did not differ between 21%O_2_ hMSCs and 1%O_2_ hMSCs (Additional file 3). Moreover, HLA-DR expression was not observed in both 21%O_2_ hMSCs and 1%O_2_ hMSCs (Additional file 3). Similarly, 21%O_2_ hMSCs and 1%O_2_ hMSCs expressed comparable levels of standard MSC markers, such as CD29, CD44, CD73, and CD90 (Additional file 3), and did not express MSC negative markers such as CD11b, CD34, and CD45 (Additional file 3).

### Conditioned medium from hypoxia-preconditioned human MSCs suppresses fibrotic changes through inhibition of TGF-β/Smad signaling

**Fig. 4:**
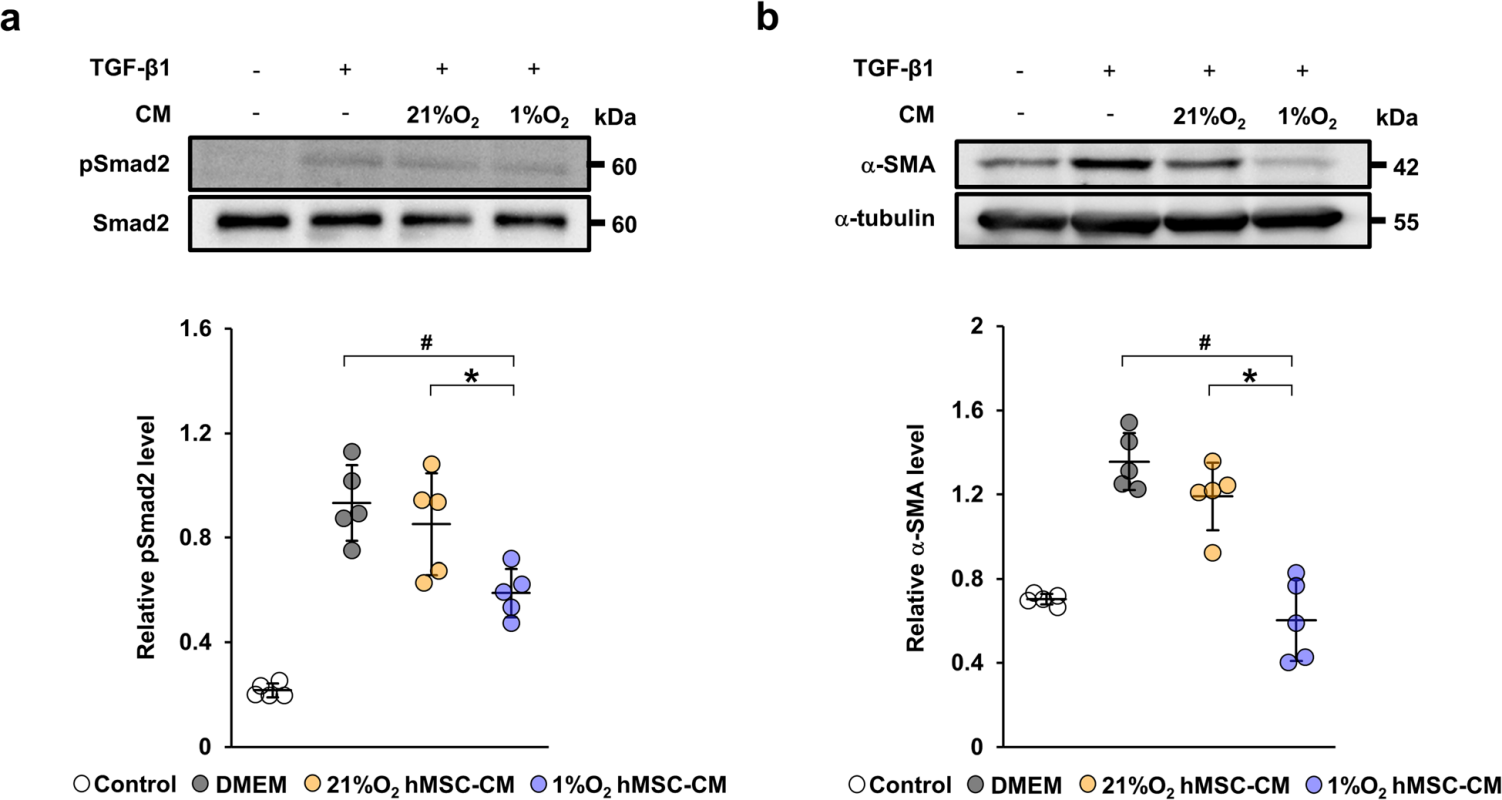
Conditioned medium from 1%O_2_ hMSCs inhibit TGF-β1-induced fibrotic changes in HK-2 cells strongly. **a** Western blot analysis of phosphorylated Smad2 (pSmad2) in HK-2 cells stimulated with TGF-β1 for 30 min. Graph shows densitometric analysis of the pSmad2 expression level normalized to the Smad2 expression level. **b** Western blot analysis of α-SMA in HK-2 cells stimulated with TGF-β1 for 48 h. Graph shows densitometric analysis of the α-SMA expression level normalized to the α-tubulin expression level. Data are means ± S.D. ^#^*P* < 0.01, *P < 0.05 (one-way ANOVA followed by Bonferroni’s post hoc test)

To identify the direct effect of hypoxia-preconditioned MSCs on TGF-β/Smad signaling, we examined the expression of phosphorylated Smad2 and α-SMA in TGF-β1-stimulated HK-2 cells. We prepared 21%O_2_ hMSC-CM and 1%O_2_ hMSC-CM, and then stimulated HK-2 cells with TGF-β1 with and without each conditioned medium. The protein level of phosphorylated Smad2 was increased by TGF-β1 stimulation. The upregulation of phosphorylated Smad2 was more significantly suppressed by 1%O_2_ hMSC-CM than both normal medium and 21%O_2_ hMSC-CM (Fig. 4a). Similar results were observed for α-SMA protein expression (Fig. 4b).

### Hypoxia-preconditioned human MSCs enhance the production of VEGF, HGF, and PGE2

**Fig. 5:**
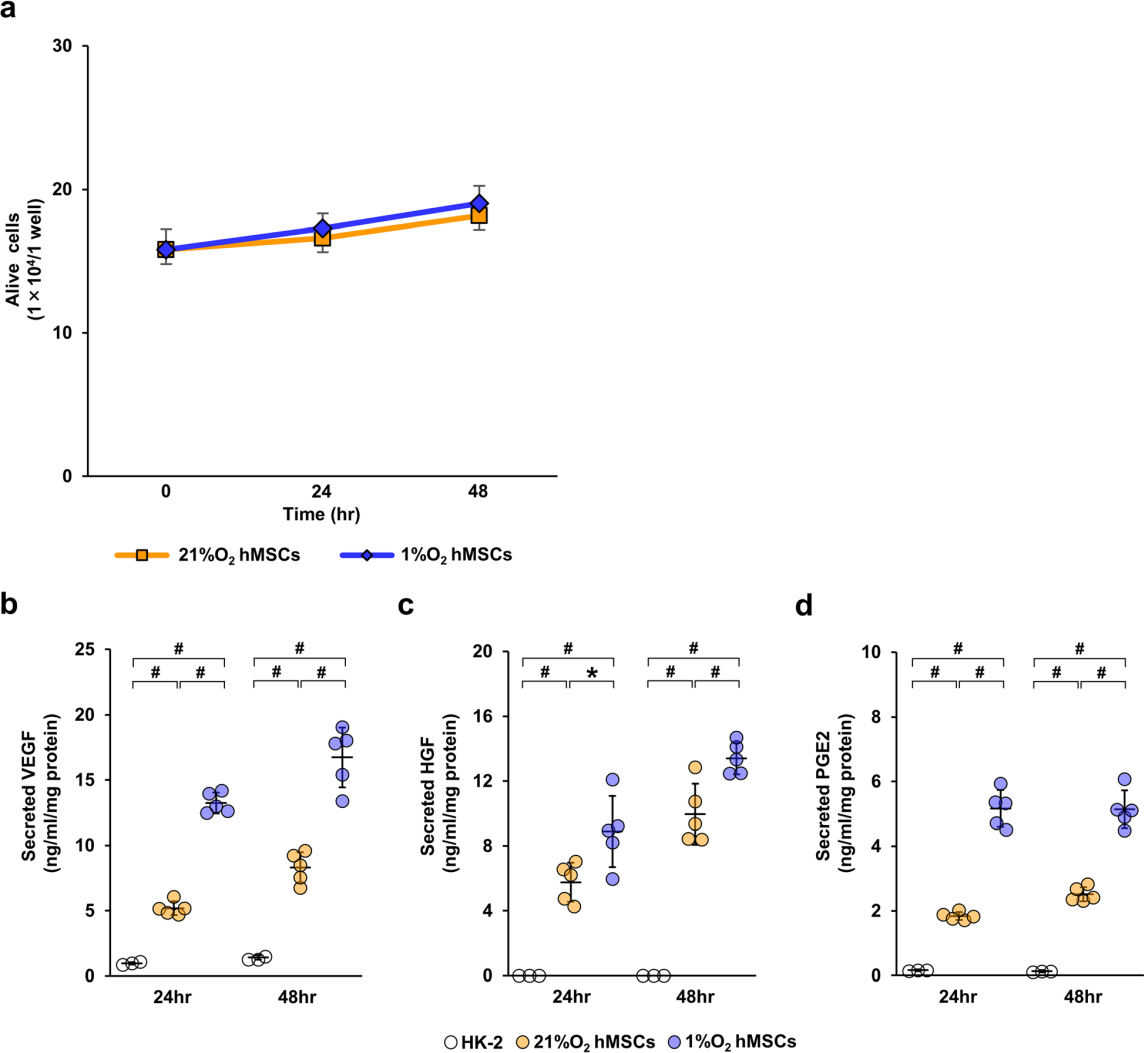
1%O_2_ hMSCs increase secretion of VEGF, HGF, and PGE2. **a** Graph showing the number of alive MSCs under normoxic conditions or 1% O_2_ conditions. Concentrations of vascular endothelial growth factor (VEGF) (**b**), hepatocyte growth factor (HGF) (**c**), and prostaglandin E2 (PGE2) (**d**) in each CM were measured using ELISA kits. Data are means ± S.D. ^#^*P* < 0.01, *P < 0.05 (one-way ANOVA followed by Bonferroni’s post hoc test)

MSCs reportedly secrete several humoral factors that promote tissue repair in a paracrine manner [22, 23]. To investigate whether hypoxia-preconditioned MSCs enhanced the secretion of these factors, we measured the concentrations of VEGF, HGF, and PGE2 in conditioned medium using ELISA kits. First, we confirmed that the numbers of both 1%O_2_ hMSCs and 21%O_2_ hMSCs were hardly increased because MSC-CM was obtained using 0.1% FBS-containing medium (Fig. 5a). As shown in Fig. 5b, we found upregulation of VEGF, HGF, and PGE2 in 1%O_2_ hMSC-CM compared with 21%O_2_ hMSC-CM. Next, we measured the expression levels of VEGF and HGF mRNA of hMSCs cultured with 10% FBS-containing medium. The number of 1%O_2_ hMSCs cultured with 10% FBS increased, as did that of 21% O_2_ hMSCs cultured with 10% FBS (Additional file 4a). In these conditions, we found upregulation of VEGF mRNA levels in the 1%O_2_ hMSCs compared with those in the 21%O_2_ hMSCs (Additional file 4b). However, there were no significant differences in the VEGF mRNA levels between hMSCs cultured under 1% O_2_ conditions for 24 h (1%O_2_ 24 h hMSCs) and 48 h (1%O_2_ 48 h hMSCs). Conversely, after both 24 and 48 h, there were no significant differences in HGF mRNA levels between 1%O_2_ hMSCs and 21%O_2_ hMSCs (Additional file 4c).

### Knockdown of VEGF in hypoxia-preconditioned human MSCs reduces the secretion of HGF and the anti-fibrotic effect in TGF-β1-stimulated HK-2 cells

**Fig. 6:**
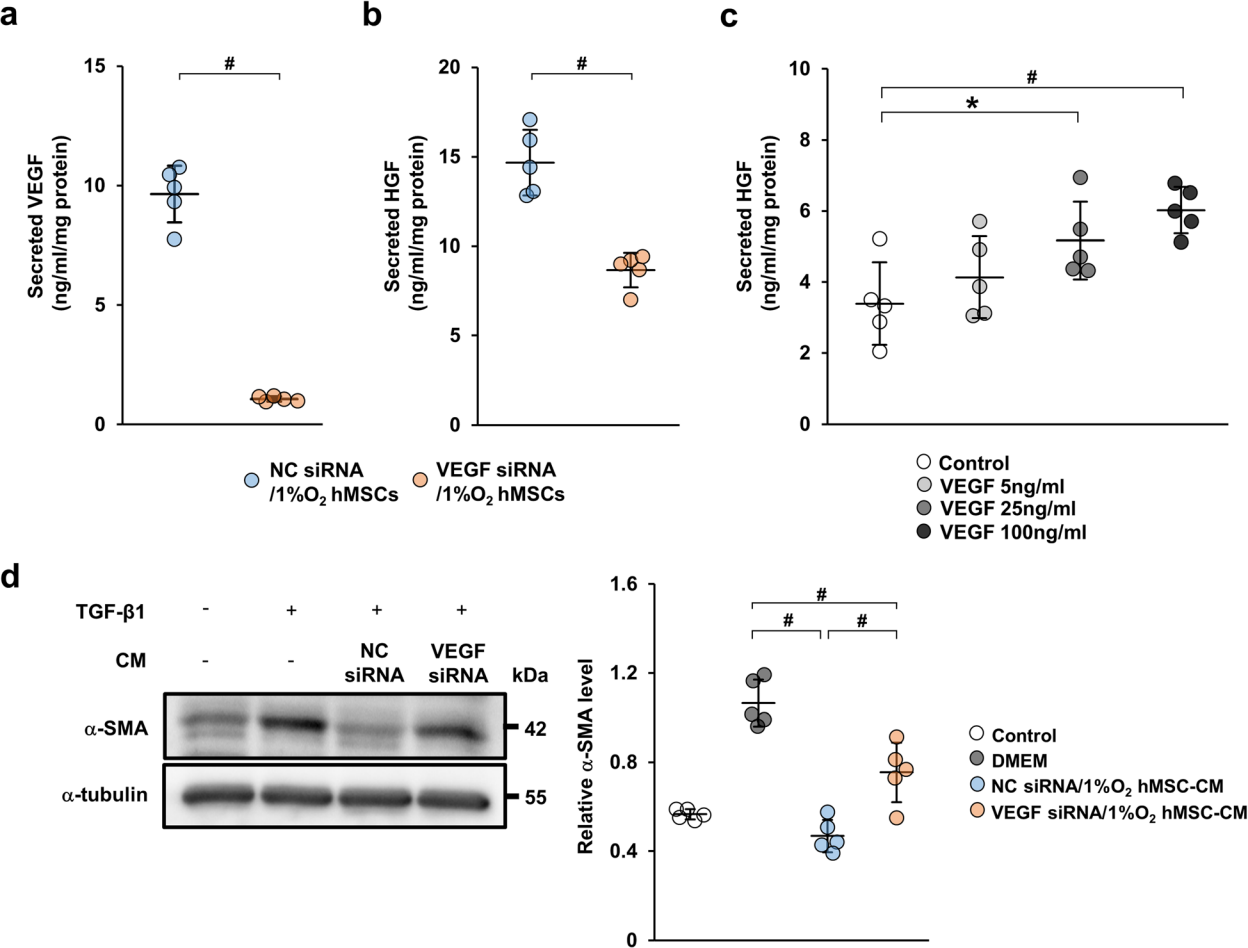
VEGF siRNA transfection of 1%O_2_ hMSCs attenuates inhibition of TGF-β1-induced fibrotic changes in HK-2 cells. 1%O_2_ hMSCs were transfected with VEGF siRNA (VEGF siRNA/1%O_2_ hMSCs) or negative control siRNA (NC siRNA/1%O_2_ hMSCs). Concentrations of VEGF (**a**) and HGF (**b**) were measured in CM from VEGF siRNA/1%O_2_ hMSCs and NC siRNA/1%O_2_ hMSCs using ELISA kits. **c** ELISA analysis showing the concentration of HGF in CM from 21%O_2_ hMSCs stimulated by VEGF at various concentrations (time, 72 h). **d** Western blot analysis of α-SMA in HK-2 cells stimulated with TGF-β1 for 48 h. Graph shows densitometric analysis of the α-SMA expression level normalized to the α-tubulin expression level. Data are means ± S.D. ^#^*P* < 0.01, *P < 0.05 (one-way ANOVA followed by Bonferroni’s post hoc test or Student’s *t* test)

The expression level of VEGF in 1%O_2_ hMSCs-CM was higher than in 21%O_2_ hMSCs-CM. Therefore, to assess whether VEGF in 1%O_2_ hMSCs-CM had an anti-fibrotic effect, we investigated 1%O_2_ hMSCs transfected with VEGF siRNA (VEGF siRNA/1%O_2_ hMSCs) or negative control siRNA (NC siRNA/1%O_2_ hMSCs). We confirmed the successful knockdown of VEGF in siRNA-transfected 1%O_2_ hMSCs by analyzing their conditioned medium (Fig. 6a). We next investigated the expression of HGF in the condition medium. As shown in Fig. 6b, ELISA analysis revealed that the expression of HGF was lower in conditioned medium from VEGF siRNA/1%O_2_ hMSCs (VEGF siRNA/1%O_2_ hMSC-CM) compared with that from NC siRNA/1%O_2_ hMSCs (NC siRNA/1%O_2_ hMSC-CM). Therefore, we examined whether VEGF was the upstream effector of HGF in hMSCs. As shown in Fig. 6c, we found that VEGF increased HGF expression after 72 h in a dose-dependent manner. Moreover, to elucidate the effect of VEGF on TGF-β1 signaling, we investigated the effect of NC siRNA/1%O_2_ hMSC-CM and VEGF siRNA/1%O_2_ hMSC-CM on α-SMA expression in TGF-β1-stimulated HK2 cells. NC siRNA/1%O_2_ hMSC-CM suppressed TGF-β1-induced the expression of α-SMA, whereas VEGF siRNA/1%O_2_ hMSC-CM attenuated this inhibitory effect (Fig. 6d).

### Knockdown of VEGF in hypoxia-preconditioned human MSCs reduces the anti-fibrotic effect in IRI rats

**Fig. 7:**
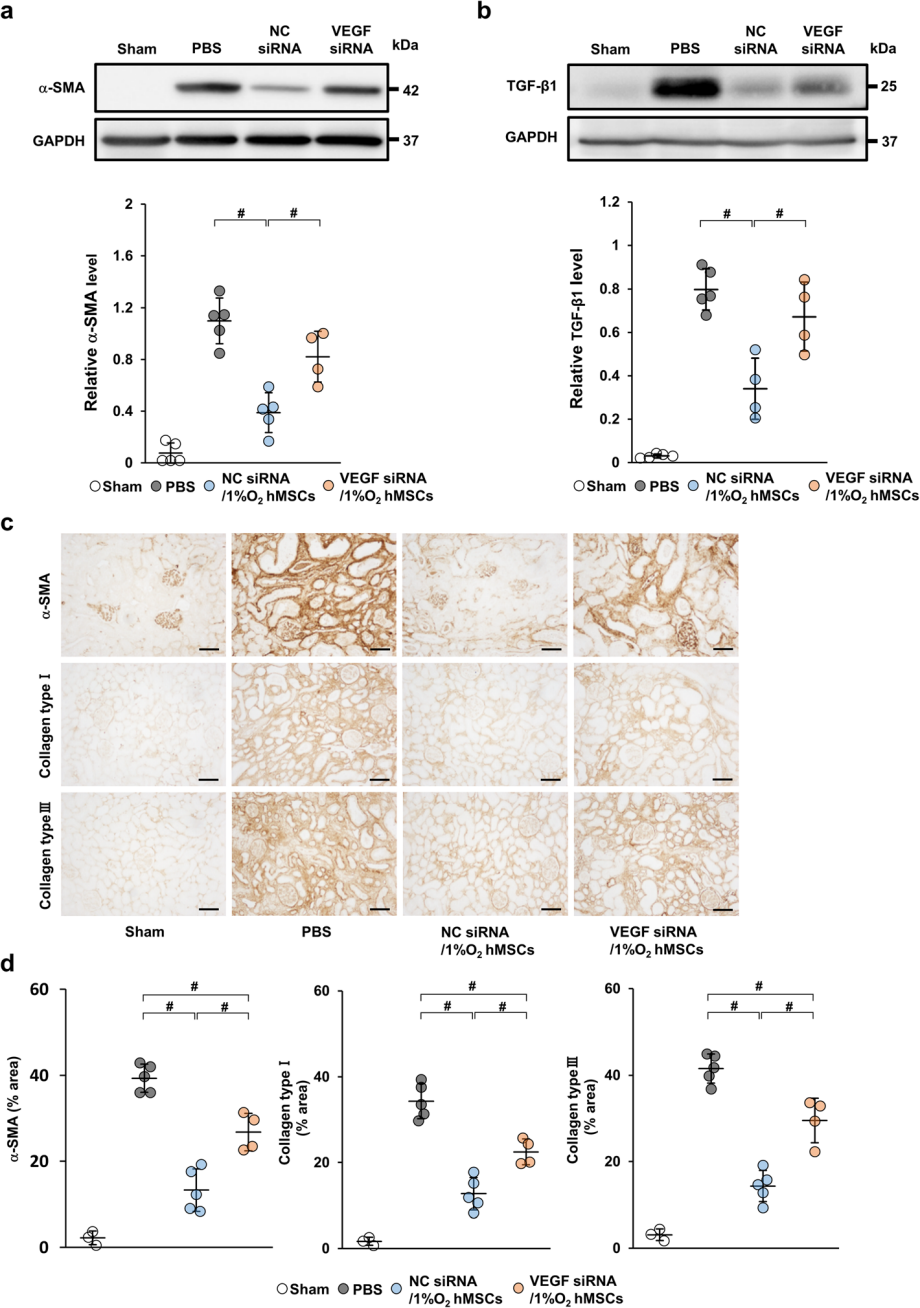
VEGF siRNA transfection attenuates the anti-fibrotic effect of 1%O_2_ hMSCs in IRI rats. **a**, **b** Western blot analysis of α-SMA and TGF-β1 in the kidney cortex of IRI rats at day 21 post-IRI. Graphs show densitometric analysis of α-SMA and TGF-β1 expression levels normalized to the GAPDH expression level. **c** Representative immunohistochemical staining of α-SMA and collagen type I and III in kidney sections at day 21 post-IRI (scale bar = 100 μm). **d** Quantification of α-SMA and collagen type I- and III-positive areas as percentages of the total area. Data are means ± S.D. ^#^*P* < 0.01 (one-way ANOVA followed by Bonferroni’s post hoc test)

To assess the effect of VEGF from 1%O_2_ hMSCs on renal fibrosis, we injected 1%O_2_ hMSCs transfected with VEGF siRNA or negative control siRNA into IRI rats (VEGF siRNA/1%O_2_ hMSC and NC siRNA/1%O_2_ hMSC groups, respectively). As shown in Fig. 7a and b, the protein levels of α-SMA and TGF-β1 were markedly increased in the PBS group and their upregulation was significantly inhibited in the NC siRNA/1%O_2_ hMSC group. However, their beneficial effect was weakened in the VEGF siRNA/1%O_2_ hMSC group. Immunostaining also revealed that the α-SMA-positive area was significantly reduced in the NC siRNA/1%O_2_ hMSC group, whereas it was decreased in the VEGF siRNA/1%O_2_ hMSC group (Fig. 7c, d). Similarly, collagen type I- and III-positive areas were markedly suppressed in the NC siRNA/1%O_2_ hMSC group, whereas the anti-fibrotic effect was reduced in the VEGF siRNA/1%O_2_ hMSC group (Fig. 7c, d).

## Discussion

This study has revealed that the administration of 1%O_2_ MSCs ameliorates renal fibrosis and inflammation in IRI rats. These beneficial effects are almost equivalent for rat- and human-derived bone marrow MSCs. In comparison with 21%O_2_ MSCs, 1%O_2_ MSCs further inhibited TGF-β/Smad signaling. Furthermore, 1%O_2_ MSCs enhanced VEGF secretion, whereas knockdown of VEGF reduced the anti-fibrotic effect of 1%O_2_ MSCs. These findings suggest that hypoxia-preconditioned MSCs enhance renoprotective effects by intensifying their paracrine abilities.

Previous studies have demonstrated that hypoxia-preconditioned MSCs attenuate tissue damage, which is mostly evaluated at the acute phase after tissue injury, but not at the chronic phase [28–30]. Pathologically, IRI induces acute tubular necrosis that follows the production of cytokines, including TGF-β1, which plays a major role in the progression of chronic renal damage [14–16]. These findings suggest that the therapeutic effects of 1%O_2_ MSCs are the result of ameliorating AKI. However, our data also showed that 1%O_2_ MSCs enhanced the ability to produce anti-fibrotic and anti-inflammatory humoral factors and that medium from 1%O_2_ MSCs inhibited TGF-β1-induced fibrotic changes. Moreover, we found that MSCs existed in the kidney at 21 days after IRI. Taken together, hypoxia-preconditioned MSCs confer the ability to suppress chronic tissue damage after AKI.

As a mechanism by which MSCs exert an anti-fibrotic effect, we previously found that conditioned medium from MSCs inhibits TGF-β/Smad signaling [31]. The presenting data showed that conditioned medium from 1%O_2_ MSCs further suppressed TGF-β1-induced phosphorylation of Smad2 and α-SMA in HK-2 cells compared with 21%O_2_ MSCs. In terms of inflammation, we found that 1%O_2_ MSCs enhanced immunosuppressive abilities in IRI rats. Among humoral factors, PGE2 is reportedly secreted from MSCs, playing an important role in changing the phenotype of macrophages from proinflammatory M1 to immunosuppressive M2 [32]. Indeed, we demonstrated that 1%O_2_ MSCs enhanced PGE2 secretion and that the administration of 1%O_2_ MSCs not only suppressed the infiltration of inflammatory cells, but also increased M2 macrophages in IRI rats. Considering that chronic inflammation is well recognized to participate in the progression of renal fibrosis [11, 14–16], these results suggest that, in addition to the inhibition of TGF-β/Smad signaling, the enhanced immunosuppressive abilities of hypoxia-preconditioned MSCs are involved in both anti-inflammatory and anti-fibrotic effects.

We demonstrated that 1%O_2_ MSCs promoted the production of VEGF and that VEGF knockdown reduced the anti-fibrotic effect in IRI rats. To adapt to hypoxic conditions, the upregulation of hypoxia-inducible factor is responsible for gene expression of VEGF, erythropoietin, and glucose transporter 1 [33–35]. Several studies have reported that VEGF functions as a renoprotective humoral factor [36–38], with evidence indicating that the administration of VEGF suppresses renal fibrosis [39]. These findings suggest that the upregulation of VEGF is implicated in the anti-fibrotic effect of 1%O_2_ MSCs. Interestingly, we identified HGF as a downstream effector of VEGF in 1%O_2_ MSCs. Previous studies have shown that HGF is a potent anti-fibrotic cytokine that antagonizes TGF-β/Smad signaling [40, 41]. Moreover, we previously found that a HGF-neutralizing antibody diminishes the anti-fibrotic effect of MSCs [31]. These results suggest that 1%O_2_ MSCs enhance HGF secretion through the upregulation of VEGF, leading to their anti-fibrotic effect. In this study, we observed that HGF mRNA levels were almost equal in 1%O_2_ hMSCs and 21%O_2_ hMSCs. Nevertheless, 1%O_2_ hMSCs showed enhanced HGF secretion. Previous studies also showed that hypoxic preconditioning enhanced HGF secretion from bone marrow-derived MSCs [24, 42]. In contrast, two recent studies reported that hypoxia-preconditioned adipose-derived MSCs exhibited a reduction of such secretion [43, 44]. These differences may be attributable to the tissues from which the MSCs were derived.

It has been reported that MSCs are characterized by the low expression of major histocompatibility complex (MHC) class I and no expression of MHC class II. Therefore, MSCs do not activate allogeneic lymphocytes [45]. Recent studies have reported that MSCs preconditioned by several inflammatory cytokines have therapeutic effects [46–48]. However, according to other studies, some cytokines upregulate MHC expression levels in MSCs, thereby elevating their immunogenicity [49, 50]. These findings raise the possibility that cytokine-preconditioned MSCs cause allogeneic immune rejection in transplantation. In this study, 1%O_2_ MSCs did not change HLA expression. AKI often occurs within a few hours or a day, and it is impossible to prepare autologous MSCs beforehand for therapy. Therefore, we attempted to demonstrate that allogeneic hypoxia-preconditioned MSC transplantation is available for treating AKI to CKD progression. For the clinical application of human MSCs, we needed to investigate whether hypoxic preconditioning enhances the anti-fibrotic ability of human MSCs to the same extent as rat MSCs, so we administered not only 1%O_2_ rMSCs, but also 1%O_2_ hMSCs to a rat IRI model. Actually, we observed that the anti-fibrotic effect of 1%O_2_ MSCs was almost equivalent in MSCs derived from rats and humans. Moreover, rat- and human-derived MSCs administered through the abdominal aorta were both observed in the kidney at day 21 post-IRI. These results suggest that hypoxia-preconditioned human MSCs have low immunogenicity and may be a good candidate for cell therapy by allogeneic transplantation. Meanwhile, Avivar-Valderas et al. reported that patients showing preexisting immunity were prone to generating donor-specific antibodies (DSA) when they were administered with allogeneic adipose MSCs. Their results suggest that sensitization may be a significant concern when patients are receiving re-treatment or multi-donor trials, although DSA generation did not correlate with MSC therapeutic efficacy [51]. Our study suggests that initial allogeneic hypoxia-preconditioned MSC transplantation has the potential to be a useful therapy for preventing the progression of AKI to CKD. However, further studies are needed to determine whether re-administration of allogeneic MSCs is available for treating AKI to CKD progression.

## Conclusions

In summary, the administration of 1%O_2_ MSCs significantly ameliorated renal fibrosis and inflammation in IRI rats compared with 21%O_2_ MSCs. We also found that 1%O_2_ MSCs confer the ability to upregulate humoral factors including VEGF, HGF, and PGE2. Among them, VEGF plays an important role in not only HGF production, but also the 1%O_2_ MSC-mediated anti-fibrotic effect. Our results indicate that hypoxia-preconditioned MSCs are useful as a cell therapy by allogeneic transplantation to prevent progression of AKI to CKD.

## Supplementary information

**Additional file 1.** Only slight interstitial fibrosis occurs at 7 days post-IRI. Representative images of HE, Masson trichrome, and Sirius red staining in kidney sections at 7 days post-IRI (scale bar = 100 μm).

**Additional file 2. **Human and rat MSCs localize in the kidney by day 21 post-IRI. MSCs collected from enhanced green fluorescent protein (EGFP)-expressing rats or DiI-labeled human MSCs were injected through the abdominal aorta after reperfusion. **a** Representative immunohistochemical staining of EGFP-positive cells (arrows) in the kidney cortex at 21 days post-IRI (scale bar = 100 μm). **b** Representative images showing DiI-labeled human MSCs (arrowheads; scale bar = 100 μm). The right panel shows periodic acid-Schiff (PAS) staining in the same tissue section (scale bar = 100 μm).

**Additional file 3.** Hypoxic preconditioning does not change expression of MSC surface markers. Flow cytometry showing expression of surface markers on 21%O_2_ hMSCs and 1%O_2_ hMSCs.

**Additional file 4. **1%O_2_ hMSCs increase the expression of VEGF mRNA, but not HGF mRNA. **a** Graph showing the number of alive MSCs cultured in medium containing 10% FBS under normoxic conditions or 1% O_2_ conditions. VEGF (**b**) and HGF (**d**) mRNA expression levels of MSCs were measured by PCR analysis. Data are means ± S.D. ^#^*P* < 0.01, *P < 0.05 (one-way ANOVA followed by Bonferroni’s post-hoc test or Student’s t-test).

## Data Availability

The data that support the findings of this study are available from the corresponding author upon reasonable request.

## Abbreviations

AKI: Acute kidney injury
CKD: Chronic kidney disease
CM: Conditioned medium
EGFP: Enhanced green fluorescent protein
EMT: Epithelial-mesenchymal transition
HGF: Hepatocyte growth factor
HLA: Human leukocyte antigen
IRI: Ischemia-reperfusion injury
MHC: Major histocompatibility complex
MSC: Mesenchymal stem cells
1%O_2_ MSCs: MSCs cultured under 1% O_2_ conditions
21%O_2_ MSCs: MSCs cultured under normoxic conditions
1%O_2_ hMSCs: Human MSCs cultured under 1% O_2_ conditions
1%O_2_ hMSCs-CM: Conditioned medium from human 1%O_2_ MSCs
21%O_2_ hMSCs: Human MSCs cultured under normoxic conditions
21%O_2_ hMSCs-CM: Conditioned medium from human 21%O_2_ MSCs
1%O_2_ rMSCs: Rat MSCs cultured under 1% O_2_ conditions
21%O_2_ rMSCs: Rat MSCs cultured under normoxic conditions
NC siRNA/1%O_2_ hMSCs: 1%O_2_ hMSCs transfected with negative control siRNA
NC siRNA/1%O_2_ hMSC-CM: Conditioned medium from NC siRNA/1%O_2_ hMSCs
PGE2: Prostaglandin E2
SD: Sprague-Dawley
TGF: Transforming growth factor
VEGF: Vascular endothelial growth factor
VEGF siRNA/1%O_2_ hMSCs: 1%O_2_ hMSCs transfected with VEGF siRNA
VEGF siRNA/1%O_2_ hMSC-CM: Conditioned medium from VEGF siRNA/1%O_2_ hMSCs
