# Supplementary material for: Hypoxia-preconditioned mesenchymal stem cells prevent renal fibrosis and inflammation in ischemia-reperfusion rats
Source: Stem Cell Res Ther. 2020 Mar 20;11:130. doi: 10.1186/s13287-020-01642-6 (PMC7083035; doi:10.1186/s13287-020-01642-6)
Supplement: Supplementary file 1 — Additional file 1. Only slight interstitial fibrosis occurs at 7 days post-IRI. Representative images of HE, Masson trichrome, and Sirius red staining in kidney sections at 7 days post-IRI (scale bar = 100 μm). [file 13287_2020_1642_MOESM1_ESM.docx]

**
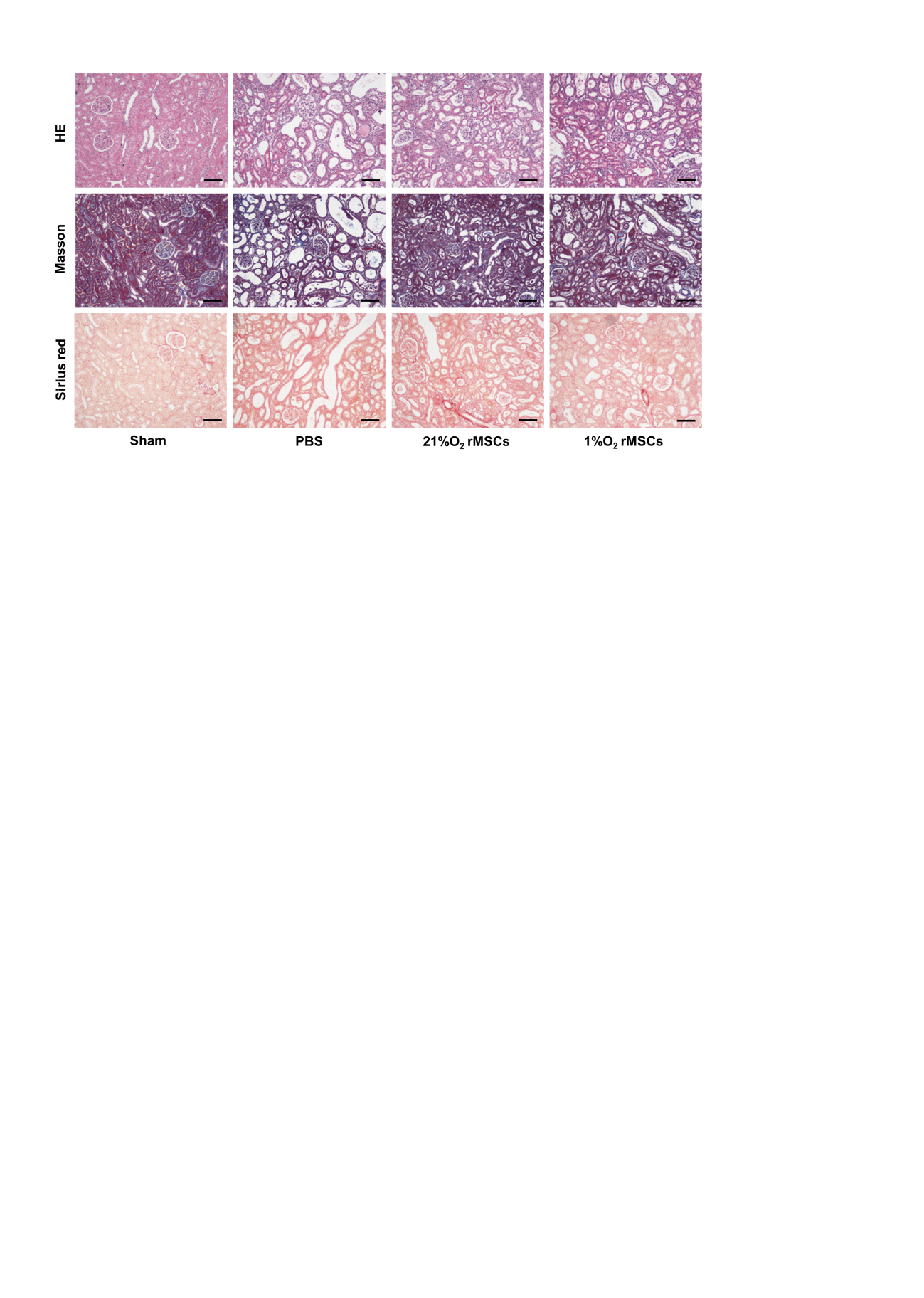
**

**Additional file 1.** Only slight interstitial fibrosis occurs at 7 days post-IRI.

Representative images of HE, Masson trichrome, and Sirius red staining in kidney sections at 7 days post-IRI (scale bar = 100 μm).
