# Supplementary material for: Hypoxia-preconditioned mesenchymal stem cells prevent renal fibrosis and inflammation in ischemia-reperfusion rats
Source: Stem Cell Res Ther. 2020 Mar 20;11:130. doi: 10.1186/s13287-020-01642-6 (PMC7083035; doi:10.1186/s13287-020-01642-6)
Supplement: Supplementary file 2 — Additional file 2. Human and rat MSCs localize in the kidney by day 21 post-IRI. MSCs collected from enhanced green fluorescent protein (EGFP)-expressing rats or DiI-labeled human MSCs were injected through the abdominal aorta after reperfusion. a Representative immunohistochemical staining of EGFP-positive cells (arrows) in the kidney cortex at 21 days post-IRI (scale bar = 100 μm). b Representative images showing DiI-labeled human MSCs (arrowheads; scale bar = 100 μm). The right panel shows periodic acid-Schiff (PAS) staining in the same tissue section (scale bar = 100 μm). [file 13287_2020_1642_MOESM2_ESM.docx]

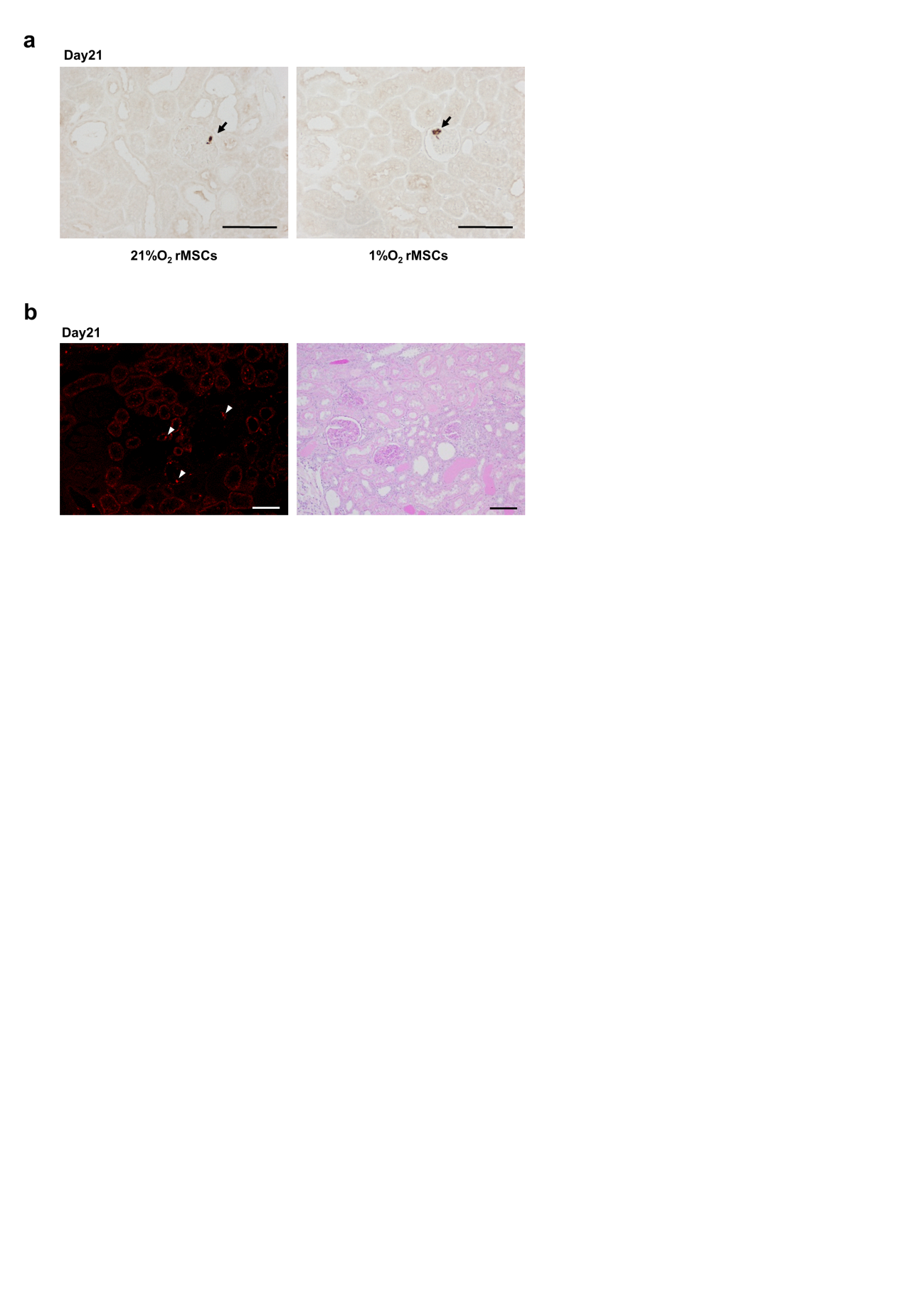


**Additional file 2.** Human and rat MSCs localize in the kidney by day 21 post-IRI.

MSCs collected from enhanced green fluorescent protein (EGFP)-expressing rats or DiI-labeled human MSCs were injected through the abdominal aorta after reperfusion. **a** Representative immunohistochemical staining of EGFP-positive cells (arrows) in the kidney cortex at 21 days post-IRI (scale bar = 100 μm). **b** Representative images showing DiI-labeled human MSCs (arrowheads; scale bar = 100 μm). The right panel shows periodic acid-Schiff (PAS) staining in the same tissue section (scale bar = 100 μm).
