# Supplementary material for: Hypoxia-preconditioned mesenchymal stem cells prevent renal fibrosis and inflammation in ischemia-reperfusion rats
Source: Stem Cell Res Ther. 2020 Mar 20;11:130. doi: 10.1186/s13287-020-01642-6 (PMC7083035; doi:10.1186/s13287-020-01642-6)
Supplement: Supplementary file 3 — Additional file 3. Hypoxic preconditioning does not change expression of MSC surface markers. Flow cytometry showing expression of surface markers on 21%O2 hMSCs and 1%O2 hMSCs. [file 13287_2020_1642_MOESM3_ESM.docx]

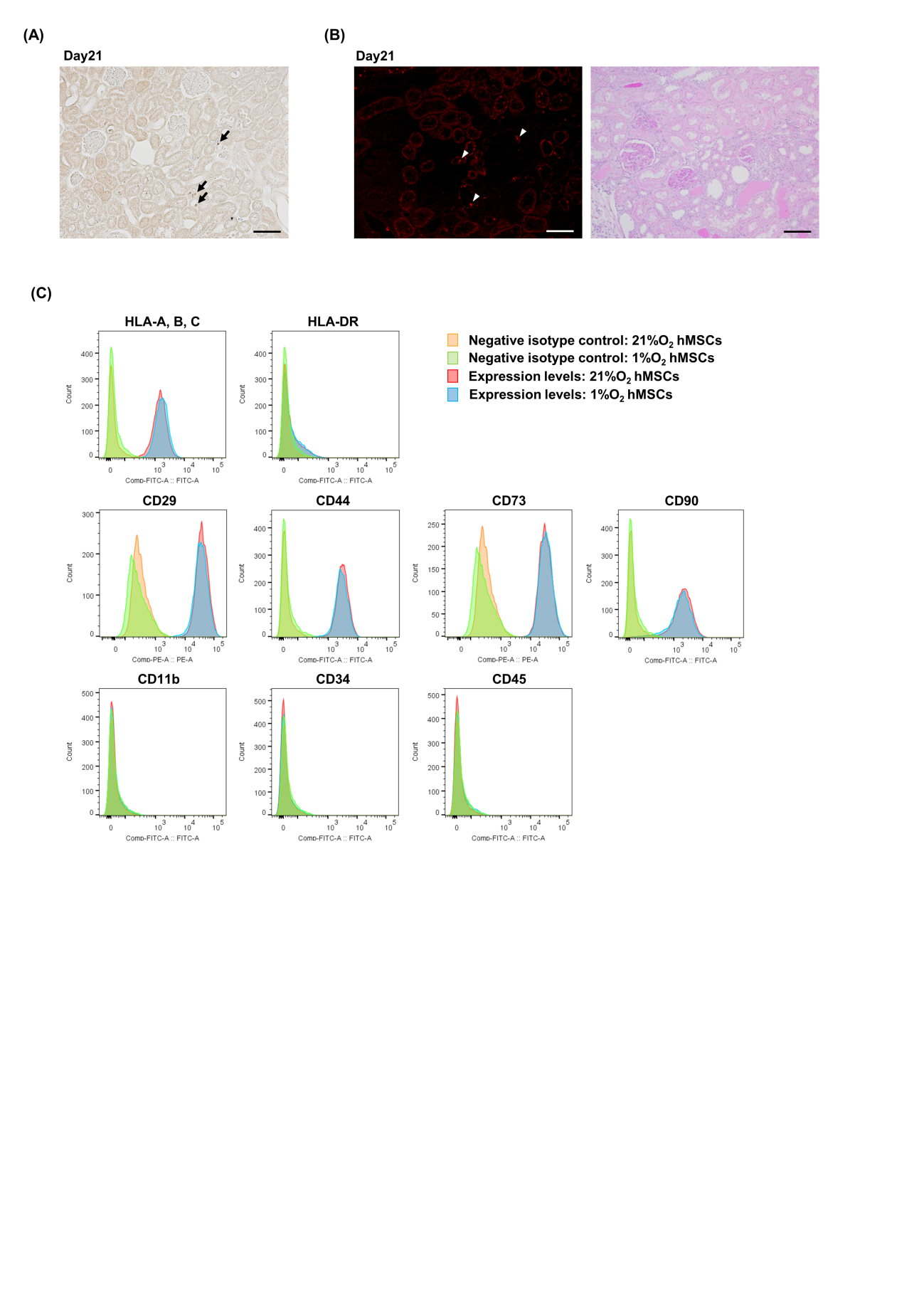


**Additional file 3.** Hypoxic preconditioning does not change expression of MSC surface markers.

Flow cytometry showing expression of surface markers on 21%O_2_ hMSCs and 1%O_2_ hMSCs.
