# Supplementary material for: Hypoxia-preconditioned mesenchymal stem cells prevent renal fibrosis and inflammation in ischemia-reperfusion rats
Source: Stem Cell Res Ther. 2020 Mar 20;11:130. doi: 10.1186/s13287-020-01642-6 (PMC7083035; doi:10.1186/s13287-020-01642-6)
Supplement: Supplementary file 4 — Additional file 4. 1%O2 hMSCs increase the expression of VEGF mRNA, but not HGF mRNA. a Graph showing the number of alive MSCs cultured in medium containing 10% FBS under normoxic conditions or 1% O2 conditions. VEGF (b) and HGF (d) mRNA expression levels of MSCs were measured by PCR analysis. Data are means ± S.D. #P < 0.01, *P < 0.05 (one-way ANOVA followed by Bonferroni’s post-hoc test or Student’s t-test). [file 13287_2020_1642_MOESM4_ESM.docx]

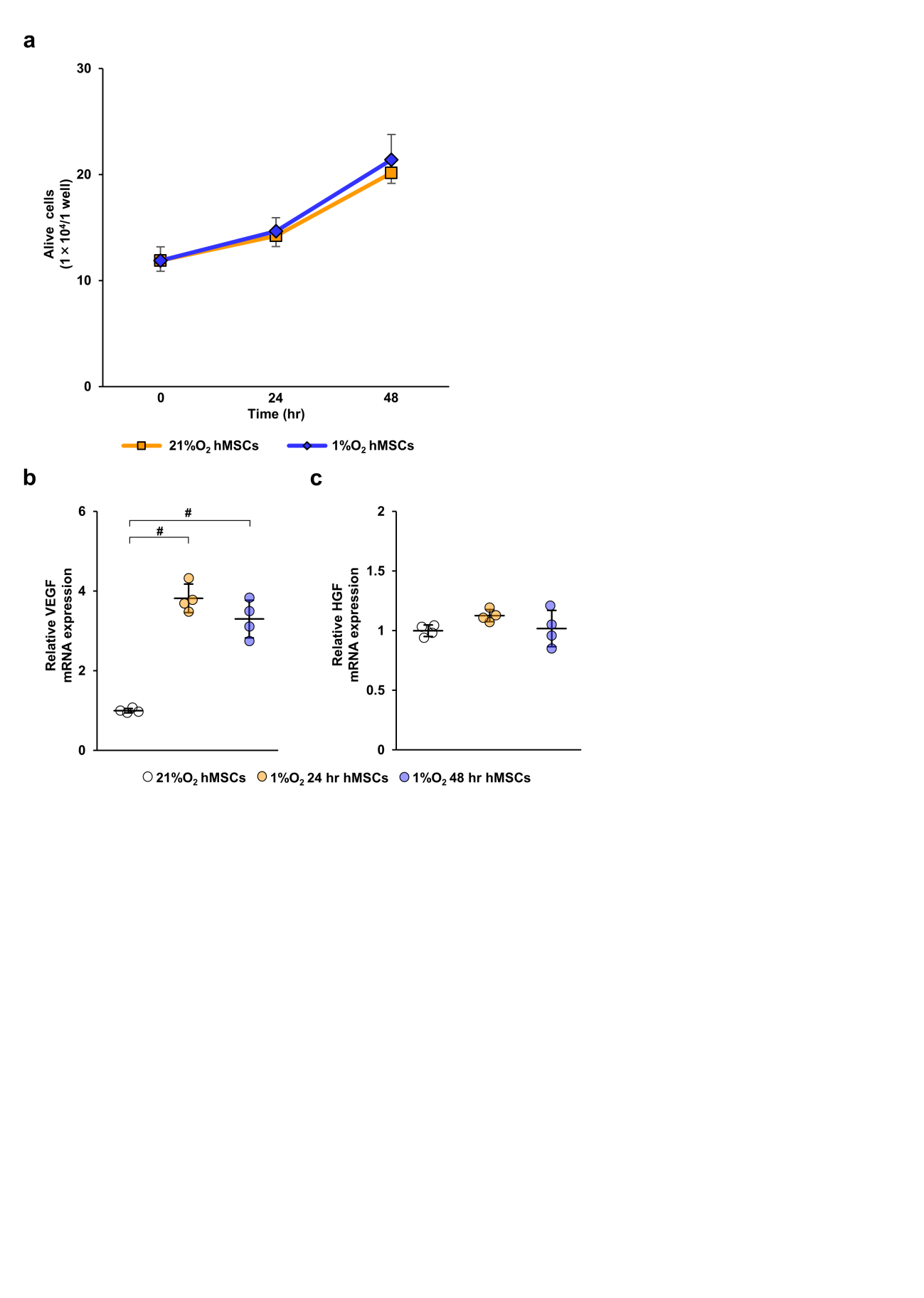


**Additional file 4.** 1%O_2_ hMSCs increase the expression of VEGF mRNA, but not HGF mRNA.

**a** Graph showing the number of alive MSCs cultured in medium containing 10% FBS under normoxic conditions or 1% O_2_ conditions. VEGF **(b)** and HGF **(d)** mRNA expression levels of MSCs were measured by PCR analysis. Data are means ± S.D. ^#^*P* < 0.01, **P* < 0.05 (one-way ANOVA followed by Bonferroni’s post-hoc test or Student’s t-test).
